# Comprehensive Care Plan Development Using Resident Assessment Instrument Framework: Past, Present, and Future Practices

**DOI:** 10.3390/healthcare3041031

**Published:** 2015-10-26

**Authors:** Mary Ellen Dellefield, Kirsten Corazzini

**Affiliations:** 1 Hahn School of Nursing and Health Sciences, University of San Diego, San Diego, CA 92110, USA; 2 VA San Diego Healthcare System, 3350 La Jolla Village Drive, San Diego, CA 92161, USA; 3 School of Nursing, Duke University, Durham, NC 27708, USA; E-Mail: Kirsten.corazzini@duke.edu

**Keywords:** resident assessment instrument, nursing home, skilled nursing facility, nursing, registered nurse, minimum data set coordinator, comprehensive care plan, quality improvement

## Abstract

Development of the comprehensive care plan (CCP) is a requirement for nursing homes participating in the federal Medicare and Medicaid programs, referred to as skilled nursing facilities. The plan must be developed within the context of the comprehensive interdisciplinary assessment framework—the Resident Assessment Instrument (RAI). Consistent compliance with this requirement has been difficult to achieve. To improve the quality of CCP development within this framework, an increased understanding of complex factors contributing to inconsistent compliance is required. In this commentary, we examine the history of the comprehensive care plan; its development within the RAI framework; linkages between the RAI and registered nurse staffing; empirical evidence of the CCP’s efficacy; and the limitations of extant standards of practices in CCP development. Because of the registered nurse’s educational preparation, professional practice standards, and licensure obligations, the essential contributions of professional nurses in CCP development are emphasized. Recommendations for evidence-based micro and macro level practice changes with the potential to improve the quality of CCP development and regulatory compliance are presented. Suggestions for future research are given.

## 1. Introduction

The Centers for Medicare and Medicaid Services (CMS) is the federal agency responsible for issuing regulatory requirements for nursing homes (NHs) participating in the federal Medicare and Medicaid NH programs, referred to as skilled nursing facilities (SNFs) [1]. The comprehensive care plan (CCP) is the document that serves as the measure of compliance with the Comprehensive Care Plans (483.20 (k)) federal requirement [2]. Surveyors determine a SNF’s degree of compliance with this requirement during standardized inspections. If practices are determined to be substandard, a citation deficiency is issued. The SNF staff must then develop a plan of correction to demonstrate their capacity for future compliance. Receipt of a citation deficiency for this and other federal requirements is significant because consumers, researchers, and other stakeholders use it as an indicator of quality.

CMS has defined the CCP as the essential communication tool to be used by the interdisciplinary team to provide coordinated services. The overall care plan orientation recommended by CMS is displayed in Table 1. Most broadly, the CCP must describe the means by which a resident may attain or maintain his highest practicable physical, mental, and psychosocial well-being while receiving SNF services. The care plan must contain quantifiable objectives and measurable outcomes. Qualified staff that provides each service must be identified. Care activities must be based on professional standards of practice tailored to the resident’s care preferences, needs, and strengths.

**Table 1:** Resident Assessment Instrument (RAI) version 3.0 overall care plan orientation [1].

Prevent avoidable declines in functioning or functional level. Managing risk factors to the extent possible. Addressing ways to preserve and build upon resident strengths. Applying current standards of practice in the care planning process. Evaluate care with measurable objectives, timetables, and care outcomes for the resident. Respect resident’s right to decline treatment. Offer alternative treatments as applicable. Use an interdisciplinary approach. Involve the resident, family, and other resident representatives. Assessing and planning care for resident’s medical, nursing, mental, and psychosocial needs. Involve direct care staff with care planning process. Address additional issues relevant to meeting the resident’s needs. (Page 4–11).

In spite of the declarations of CCP’s essential functions by CMS, consistent CCP development has been difficult to achieve. For example, researchers reported that over the seven year period of 1994 to 2000, 17% to 29% of citation deficiencies for substandard care targeted non-compliances with use of CCPs [3]. Two Office of Inspector General (OIG) reports found evidence of a significant number of SNF failures to comply with regulations regarding care plans [4,5]. ProPublica’s Nursing Home Inspect (2012, 2013) reported that the 5th of the top 10 citation deficiencies nationally was failure to develop CCPs [6].

Complex factors have influenced how standards of practice for CCP development have evolved. In this commentary, we examine these factors. They include: the history of the comprehensive care plan; its development within the RAI framework; linkages between the RAI and registered nurse (RN) staffing; empirical evidence of the CCP’s efficacy; and limitations of extant standards of practice used in CCP development. Because of the registered nurse’s educational preparation, professional practice standards, and licensure obligations, the essential contributions of professional nurses in CCP development are emphasized [7]. Recommendations for evidence-based micro and macro level practice changes to improve the quality of CCP development, while complying with federal requirements, are presented. Suggestions for future research are given.

## 2. Past Nursing and Regulatory History of CCPs

The care plan document played seminal roles in nursing’s advancement as an applied science, the development of nursing curricula, and implementation of the federal SNF programs [8]. A detailed history of each is available to readers [8,9]. Brief histories of key events in CCP development are displayed in Table 2 and Table 3.

**Table 2:** CCP nursing history in United States [8,9,10,11,12,13,14,15,16].

Year	Event
1900s	Care plans developed by nurse educators teaching tool; used by working student nurses.
Post WW II	Baccalaureate (BSN) education in university settings recommended for RNs. Care plan development and care planning defined as core competencies of BSN prepared RNs.
1950s	The structure of the care plan document was defined by Dr. Ida Orlando.
Post WW II–1960s	Due to persistent nursing shortages, hospitals encouraged development of non-BSN prepared nurses: nursing assistants, licensed vocational/practical nurses, associate degree, and diploma nurses.
1965	Care plan document included as a Medicare Condition of Participation (COP) participation in federal Medicare and Medicaid SNF programs.
1966	The Joint Commission on Accreditation of Hospital Organizations (JCAHO) Long Term Care Accreditation Program was initiated.
1969	The nursing care plan document became a JCAHO accreditation standard.
1987	The Omnibus Budget Reconciliation Act (OBRA) of 1987 mandated replacement of nursing care plans with interdisciplinary care plans (ICPs).
1991	The ICP was renamed as the comprehensive care plan (CCP).
2013	The Joint Commission replaced the Long Term Care Accreditation program with the Nursing Care Center (NCC) Accreditation program. This was done in response to changes in the Medicare and Medicaid reimbursement system and the role of SNFs in the post-acute care continuum.

### 2.1. Nursing History

The nursing care plan was invented in the early 1900s by nursing faculty and used as a didactic technique. Its purpose was to teach nursing students cognitive and behavioral skills required to provide holistic and individualized care to hospital patients [10]. The care plan was also a practical work tool. Student nurses used it to identify care activities to be provided around the clock, 7 days a week while working as hospital staff a common practice in the 1900s. Significantly, the nursing care plan was developed as a clinical tool for student nurses; it was never intended to be used by experienced nurses [11].

Following World War II, the nation experienced a nursing shortage. It became possible for individuals to earn nursing licenses or certifications through a variety of educational programs. This resulted in a nursing staff that had diverse licensures and educational backgrounds. The resulting mixture of different types of nursing staff was referred to as an institution’s *nursing skill mix* [12]. Because it described the composition of the nursing labor force, this skill mix significantly influenced the development of nursing care delivery systems [12]. For example, practical nurses and nursing assistants were used to provide technical or direct patient care. The RN functioned as the problem solver and team manager. Arguably, this historic bifurcation of nursing labor likely contributed to nursing’s ongoing struggle in integrating and valuing cognitive and technical nursing skills equally.

In the late 1950s, clinical problem solving referred to as the *nursing process* was codified as the unique domain of RNs. Dr. Ida Orlando, a prominent nursing researcher, defined the nursing process as: assessment, definition of problem, planning, implementation, and evaluation of care [13]. Her definition provided the standardized format for the nursing care plan document. Because the nursing care plan was based on the nursing process, the care plan evolved from its historic purpose as a tool for student nurses into a standard of practice for RNs. The nursing process and the care plan document became embedded in the curricula of baccalaureate and post-graduate nursing education [14]. RN competencies in the care planning process were integrated into state nurse practice acts, the American Nurses Association (ANA) standards of practice, and nursing curricula [13,14,15,16]. The degree to which the discipline of nursing has integrated competencies in the nursing process (e.g., clinical decision-making) and development of the care plan document into the RN’s professional identity is essential to understand [7,8].

The discipline has moved beyond its singular focus on the nursing process to describe the work of the RN. RN practice is conceptualized as dynamic; it requires the RN to think critically and made clinical decisions based on empirical evidence and the preferences of the patient [15]. However, RNs practicing in SNFs have been slow in defining their competencies in these ways. Arguably, this is a consequence of their SNF RN practice being largely prescriptive and rule-bound, as reflected in the RAI framework.

#### RAI History

Since the establishment of the SNF conditions of participation in the Medicare and Medicaid NH program, concerns were expressed about the quality of care provided in SNFs. In response, recommendations for structural and process changes in the federal government’s oversight of SNFs were published in the Institute of Medicine (IOM)’s report—*Improving the Quality of Care in Nursing Homes* [17]. A key recommendation of the IOM report was the establishment of a federally mandated standardized comprehensive clinical assessment process. The RAI was developed and introduced in1990. It was comprised of three components—the Minimum Data Set (MDS) version 1.0, a 450-item instrument linked with standardized definitions and coding instructions; the Resident Assessment Protocols (RAPS); and CMS Utilization Guidelines of the RAI [18]. The nursing care plan document was replaced by the interdisciplinary care plan (ICP).

Descriptions of the development and evolution of the MDS related to studying the reliability and validity of MDS-derived data are available [18,19]. While previously researchers have reported evidence of the reliability and validity of MDS-derived data, concerns about the reliability of MDS-derived data have been raised [20]. In response, CMS piloted special surveys of SNFs focused on verifying the accuracy of MDS 3.0 data used for quality measures [21].

Table 3 displays a brief history of the evolution of the RAI. The Balanced Budget Act (BBA) of 1997 gave CMS the authority to implement the Medicare Prospective Payment System (PPS) as the method of reimbursement for SNFs. In 1995, MDS data were used to group residents based on their characteristics and associated staff time used. They were referred to as Resource Utilization Groups, version III (RUG-III). RUG-III were first used in 10 states to categorize Medicaid beneficiaries’ nursing and therapy resource needs, and associated payment rates, adjusted for variations in regional wages [22]. RUG-III became the national method for determining SNF levels of reimbursement within the PPS [23].

**Table 3:** RAI history [17,18,19,20,21,22,23,24,25,26].

Year	Event
1986	IOM report was published.
1986	The RUG-II New York State Medicaid case-mix payment system was developed.
1990–1991	The RAI MDSwas developed and implemented
1990	The language changed from nursing to interdisciplinary care plan
1994	RUG-III development and testing was done.
1995	The RUG-III was used in 10 state Medicaid programs.
1995	MDS 2.0 was implemented. Quality Indicators (QI)s were developed and implemented.
1997	The Prospective Payment System for SNFs was implemented.
2000	MDS 2.0 revisions were made. Electronic transmission of data began in 1998 and was required by 2000.
2002	The Quality Measures (QM)s were implemented.
2010	RUG-IV was implemented.
2012	MDS 3.0 was implemented.
2012	The Resident Assessment Protocols (RAPS) were replaced with Care Area Assessments (CAAs).
2014	A component of the Accountable Care Act, (IMPACT), mandated changes in the Medicare and Medicaid reimbursement system.

Since the electronic transmission of MDS data was mandated by 2002, CMS used its technical capacity to develop quality indicators (QI) and quality measures (QM) for use in enforcement and quality improvement efforts [24,25]. The ICP was renamed as the CCP. Among other revisions made in MDS 3.0, the RAPS were replaced with Care Area Assessments (CAAs). The prescribed decision-making process that determines the development of the CCP was contained within the CAAs [26].

Researchers have used MDS data for studies in both the United States and internationally. The MDS has been translated into 11 languages (Danish, Dutch, French, Icelandic, Italian, Spanish, Swedish, German, Czech, Finnish, and Norwegian) and used in various ways in Italy, United Kingdom (UK), Japan, Iceland, Switzerland, and Czech Republic) [27,28,29,30,31]. Although nations differed in how they implemented the RAI framework overall, the MDS database was used by all for analysis of NH quality, costs, and cross-national comparisons of resident characteristics.

As this brief history demonstrates, the RAI framework is complex and dynamic. Future changes to the RAI are anticipated in response to implementation of the Improving Medicare Post-Acute Care Transformation (IMPACT) Act of 2014. New and streamlined quality measures for SNFs, home health agencies, and other post-acute providers participating in Medicare will be developed. Referred to as Standardization Resident Patient Assessment Data, this new database will enable CMS to compare quality across post-acute care settings, improve hospital and post-acute discharge planning, and to reform post-acute care payments [32].

## 3. Empirical Evidence

### 3.1. Search Strategies

After studying the history of the CCP, we conducted a “scoping literature review” to determine the amount and type of research studies and grey literature published on CCPs developed within the RAI framework. Our inclusion criteria were grey literature and research studies published in English between 1997 and 2014 and conducted in the United States or other countries that used the RAI framework. Key words included: skilled nursing facility, nursing care plan, RN practice, care planning, RAI, MDS, nursing process, nursing home, teamwork, interdisciplinary care, and comprehensive care plans. The search excluded advanced care planning related to end-of-life decision making in health care because of the specialized nature of its focus.

Study designs included systematic reviews, randomized clinical trials, quasi-experimental, descriptive, cross-sectional, qualitative, mixed methods, case studies, and program evaluations. The quality of the studies was not assessed, as would be done in a systematic review. Our sole purpose was to establish a baseline summary of studies that examined development and or use of the CCP within the RAI framework. The findings were used to provide support for recommendations made in this commentary and as background for a systematic review. Findings are summarized in Table 4, Table 5 and Table 6 [4,5,14,21,33,34,35,36,37,38,39,40,41,42,43,44,45,46,47,48,49,50,51,52,53], excluding the systematic reviews [54,55,56,57].

### 3.2. Systematic Reviews

Four systematic reviews on care plans and interdisciplinary interventions in NHs were found. They did not include quantitative analyses of study findings included in the review, as is currently done in systematic reviews. Moloney (1999) conducted a systematic review of studies that focus on the existence of a relationship between care planning and record keeping in nursing and patient outcomes [54]. Moloney determined that no studies of sufficient rigor had been conducted. Muller-Staub (2006)’s systematic review of various nursing processes associated with care plan documentation and patient outcomes reported mixed findings [55]. The author concluded that the systematic review demonstrated the lack of rigor in study designs because of the complexity of the relationships studied, and challenges with measurement methods.

**Table 4:** Studies of RAI-related structural variables [4,5,21,33,34,35,36,37,38].

Author	Setting	Sample size	Data sources	Design	Measures	Main findings
Hawes, 1997 [33]	SNFs	254 SNFs	2 resident cohorts (>2000); 10 states	Quasi-experimental probability-based sample	Completeness, accuracy-care plans; medical records	Increased medical record accuracy; Completeness of care plan
Bernabei, 1997 [34]	RAI training sites	9 countries	Staff participating in training sessions	Descriptive	RAI training sessions: purpose, length, content	Greatest variation in training between US and other countries
Hansebo, 1998 [35]	Sweden	3 elder care facilities; 50 nursing staff	Nursing staff trained in RAI	Cross-sectional survey	Staff views of RAI/MDS and care quality	Most staff reported positive improvement in care quality with RAI/MDS
Ettinger, 2000 [36]	428 Iowa SNFs	236 directors of nursing (DON)	DON surveys	Cross-sectional survey	DON perceptions of utility of dental section	76% viewed MDS section as useful; 9% used to identify dental needs
Jogerst, 2001 [37]	Geriatric MD practices	472 MDs	Internist and family MDs	Cross-sectional survey	% time reviewing MDS and CCP; how used; attitudes about MDS	11% reviewed all MDS and 21% partially. 19% did not review CCP; 56% had negative or derogatory attitudes
Parmelee, 2009 [38]	VA NH care units	289 NHs; 259 VA NH staff	34 DONs, 96 MDS RNs, 97 nurse managers; 19 medical directors; others 13	Mixed methods:	Accuracy. Usefulness, utility for quality improvement	78.4% rated as very accurate or accurate; 85.7% rated MDS as useful; 85.7% rated QIs as very or somewhat useful.
Abt report to CMS 2015 [21]	SNFs	Pilot survey SNFs	RAI/MDS documents	Retrospective descriptive	Evidence of adherence to MDS 3.0 reporting requirements, RN role; accuracy	99% compliance with mandated RN participation; 2.2% of MDS noncompliant with required timelines; MDS assessment/medical record discrepancies ranged 0.8% to 25.5%.

Shaded rows indicate studies using mixed methods.

**Table 5:** Studies of RAI-related processes variables [4,14,39,40,41,42,43,44,45,46,47,48,49,50,51,52,53].

Author	Setting	Sample Size	Data Source	Design	Measures	Main Findings
Hawes, 1997 [33]	SNFs	254 SNFs	2 resident cohorts (>2000); 10 states	Quasi-experimental probability-based sample	Use of physical restraints and indwelling catheters; Use of advanced directives; Resident participation in activities and toileting programs for bowel incontinence	Decreased use of physical restraints and indwelling catheters; Increased use of advanced directives; Increased resident participation in activities and toileting programs for bowel incontinence
Achterberg, 2001 [39]	Dutch NHs	10 NHs; 18 wards	Interviews with residents and staff	Quasi-experimental	Quality of coordination	Improvement in care coordination post RAI implementation
Lee, 2003 [40]	Midwest NHs	3 NHs	Observation, interview, medical record review	Mixed methods	Process based costing of care planning in NHs	Calculating directs costs for care planning is possible. Data collection for costs is based on a process map.
Tauton, 2004 [41]	Midwest NHs	3 NHs	Semi-structured interview, observation, chart audit	Mixed methods/case reports	Care planning process	Facilities differed in their approaches; care linked to other methods of communication and records.
Piven, 2006 [42]	SNFs	2 SNFs; 4 MDS coordinators	Staff interviews with MDS coordinators, administration, nursing social work, activities, rehabilitation, dietary, environmental services	Comparative multiple case study	MDS Coordinators’ patterns of relationships and association with care processes	Positive MDS patterns generated new information flow, good connections, cognitive diversity contributed to positive assessment and care planning. Negative MDS patterns had opposite effect
Bott, 2007 [43]	NHs in Mid-west	Random sample-107 NHs; 437 staff	Staff interviewed: MDS coordinators; assistant coordinators; social services directors, activities directors, dietary directors; other staff (medical records, LVN, therapists, nursing assistants).	Mixed methods	Process-based costing; Indicators for data envelopment analyses (DEA)	2 NHs were most efficient (fewer deficiencies, less time spent in care plan meetings); Less efficient NHs spent 2 to 5 more time in CP meetings and no increase in quality or efficiency. SNFs less likely to be efficient
Colón-Emeric, 2007 [44]	SNFs	4 SNFs; 360 staff	Field observations; shadow encounters; in-depth interviews	Comparative multiple case study	Relationship between staff connections and care planning process	Greater staff connections associated with higher care plan specificity (tailored) and innovation
Adams-Wendling, 2008 [45]	NHs in Mid-west	Purposeful sample of 96 residents’ care plans	Care plan documents	Retrospective case review	Care plan content	Translation issues included: CP length; content (routine practices and redundant interventions); variability in language use; fragmented care plan and poor location
Dellefield, 2008 [46]	AANAC national conference	24 RN MDS coordinators	Focus groups; questionnaires	Mixed methods	Description of MDS Coordinator work in organizational context	Structural, technical, cultural, strategic organizational dimensions influenced work of MDS coordinator
Taunton, 2008 [47]	NHs-Kansas, Missouri	107 random sample NHs; 508 staff members	Telephone interview, OSCAR data	Mixed methods (Correlational model generation-model selection design)	Generate empirically supported model of care planning integrity	Care planning integrity demonstrated through direct relationships with coordination, integration, quality; indirect relationships through integration with IDT team and restorative perspective.
Straker, 2008 [48]	NHs Ohio	997 NHs; 202 respondents	Stratified random sample NHs; random sample staff	Descriptive	Processes used to complete MDS	MDS process is time intensive, involves various staff, requires training, manual is valuable.
Lee, 2009 [49]	NHs-Kansas, Missouri	107 NHs; 437 staff	Staff interviews: MDS coordinators; assistant coordinators; social services directors, activities directors, dietary directors; medical records, LVN, therapists, nursing assistants	Mixed methods-Interviews and regression and DEA analyses	Efficiency of assessment process; Average cost and quality of care plan	NHs used different combinations of staff to complete care plans; Plans/week varied 10 fold; average cost varied 8 fold; 47% had no care plan deficiency in most recent survey.
Kontos, 2009 [50]	NHs in Central Canada	26 personal support workers (PSW)s; 9 supervisors	Focus groups and semi-structured interviews	Focus groups and interviews	Decision-making and care practices of PSWs in relation to RAI/MDS process	Assessment information known by PSWs not captured in RAI/MDS categories or communicated to interdisciplinary team. Factors included lack of access to computerized records, low status, and poor inter-professional collaboration
Lindsay Bratton-Mullins, 2010 [14]	Historic and current nursing text books	7 textbooks	Text in textbooks on care plan education	Phenomenological analysis	Care plan as indicator of change in nursing science instruction	Care plan development used to teach critical thinking skills to RN students
Colon-Emeric, 2010 [51]	SNFs	8 SNFs; 958 staff	Field observations; direct observation; and interviews	Content analysis of in-depth multiple-case study	Purpose and utility of regulations (including RAI/MDS)	Increased mindful behaviors in resident centered SNFs; Reduced mindful behaviors in cost-focused culture due to regulation

**Table 6:** Studies of RAI-related outcome variables [4,5,52,53].

Author	Setting	Sample size	Data source	Design	Measures	Main findings
OIG, 2012 [4]	640 SNFs	Random sample of 375 Medicare claims for atypical anti-psychotic drugs	Medical records; Documentation related to resident assessment, decision-making, care plans	Retrospective descriptive	Compliance with regulatory requirements for assessment and care plans of residents receiving atypical anti-psychotic drugs	99% of records lacked evidence of compliance with CCP requirements (including care plan development)
OIG, 2013 [5]	SNFs	Stratified random sample- 190 Medicare stays	Medical records; Documentation related to resident assessment, decision-making, care plans	Retrospective descriptive	Compliance with regulatory requirements	37% of records lacked evidence of compliance with CCP requirements (including care plan development)
Holtkamp, 2000 [52]	NHs	10 NHs; 6 experimental wards; 8 control wards; 337 residents	Resident and staff interviews; medical records	Non-randomized controlled design	Gap between resident perceived needs and nursing care received; relationship between coordination of care and care discrepancies	Perceived gaps decreased in experimental group; higher care coordination associated with fewer perceived gaps
Chi, 2010 [53]	Hong Kong NHs	10 NHs; 5 in each group; 571and 519 residents respectively	RAI/MDS data	Prospective 10 month randomized clinical trial	Effects of RAI/MDS care planning on General health of residents	No significant differences found

In *Patient Safety and Quality: An Evidence-Based Handbook for Nurses* (2008), Keenan, Yakel, Tschannen, and Mandeville (2008) reported findings of a systematic review of studies on nursing documentation, care plans, and ICPs used in acute and long term care settings that were published between 1997 and 2006 [56]. The authors concluded that studies of nursing care plans lacked rigor in their design and execution. For example, often their primary focus was on the care plan document itself. No evidence was found to support the notion that the care plan document increased continuity of care across time and space. Similarly, studies of interdisciplinary care planning lacked scientific rigor because they were primarily focused on case management or clinical pathways. The authors concluded that no empirical evidence of a significant relationship between care planning and resident outcomes was found.

Given the importance of interdisciplinary team work in the SNF, and the lack of rigor reported in earlier systematic reviews, Nazir’s (2013) systematic review of randomized controlled trials of interdisciplinary interventions was noteworthy [57]. Using formal teams in SNFs, interdisciplinary team interventions had positive impact of resident outcomes. The review included a wide range of interdisciplinary interventions that focused on CCP team meetings, medication reviews with IDT members, fall prevention, and incontinence management. Team communication and coordination were consistent features of successful interventions [57].

As the findings of the systematic reviews suggest, development of the interdisciplinary care plan and or the CCP involves complex interrelated processes, each of which has unique characteristics. Table 7 displays the 5 steps of the RAI framework.

**Table 7:** Five steps of the RAI framework [1].

Assessment (MDS) → Decision-Making (CAA) → Care Plan Development → Care Plan Implementation → Evaluation

#### 3.2.1. Findings of Scoping Review

Twenty-four studies related to care plans and interdisciplinary teams within the RAI framework were reviewed. The methods used include: quantitative [11] and qualitative methods, including mixed methods [13]. Regardless of method used, the studies are summarized in Table 4, Table 5 and Table 6 based on RAI-related variables (*i.e.*, structural, process, or outcome) measured. Studies that used mixed methods are indicated by shaded rows.

#### 3.2.2. Studies Categorized by RAI-related Variables

##### Structural Variables

The CCP document itself has been used as a structural measure of compliance. Hawes and colleagues (1997) found evidence of more comprehensive (e.g., completeness) ICPs developed within the RAI framework [33]. They reported initial positive assessment of RAI by staff that used the original version of MDS [33]. Other measures of structure related to the RAI included differences in MDS training programs [34], staff attitudes about quality and the RAI [35,36], utility [37], accuracy of MDS data [21,38], and failure to develop CCPs [4,5].

In *Nursing Facility Assessments and Care Plans for Residents Receiving Atypical Antipsychotic Drugs (*2012), analysts found that 99% of randomly sampled records did not comply with federal resident assessment and/or care plan requirements [4]. In *Skilled Nursing Facilities Often Fail to Meet Care Planning and Discharge Planning Requirements* (2013), researchers found that 37% of NH stays lacked documentation of care plans based on a stratified simple random sample of 2009 SNF medical records [5].

##### Process Variables

Characteristics of processes measured included: use of physical restraints and indwelling urinary catheters; use of toileting programs for residents with bowel incontinence; rates functional decline, and good care practices [33], care coordination post RAI implementation [39], care planning costs [40], approaches to implementation and communication among staff [41], MDS coordinator patterns of relationships related to care planning processes [42], time spent care planning [43], staff connections [44], characteristics of CCP content related to routine practices [45], the factors affecting performance of RN MDS coordinator [46], the integrity of the care planning process [47], types of staff used to complete CCP [48,49], information exchange between direct care workers and interdisciplinary team members [50], use of care plan development in nursing curricula [14], and staff understanding of practice within the regulatory framework [51].

Findings provide support for a relationship between management practices and culture and CCP development and implementation. Interestingly, the practices that promote innovative and valued CCP development are also those that promote effective team work, safe work cultures [58,59,60], and effective information exchange among SNF staff [61]. For example, safety-focused programs, such as TeamSTEPPS^®^ Long Term Care Version [60] and culture change programs focus on development of these organizational characteristics [62].

##### Outcome variables

Several outcomes were measured to assess efficacy of the RAI framework on clinical outcomes. Hawes and colleagues (1997) found increased resident participation in activities and use of advanced directives, after controlling for differences in resident characteristics between 1990 and 1993 [33]. The gap between the resident’s perceived needs and quality of care was lower in the Dutch NHs that implemented the RAI *versus* the control group [52]. In a randomized controlled trail, Chi did not find significant differences in outcomes between the experimental group that implemented the RAI and the control group [53] significant differences in outcomes between the experimental group that implemented the RAI and the control group [53].

## 4. Present Practices

Every SNF must develop a CCP within the RAI framework. The CCP document is either hand-written or electronically produced using MDS-related software [63]. As previously noted, the specific staff members participating in CCP development and meetings are not prescribed. The CCP must include quantifiable objectives and measureable outcomes; identification of the staff performing the activity; and must be based on extant professional standards of practice that have been modified to address a resident’s care preferences, needs, and strength.

CMS provides standardized instructions for different components of the framework. Definition of terms, coding rules, and standardized timelines for acquisition and electronic transmission of MDS 3.0 data are provided in the Utilization Guidelines. For example, the prescribed decision-making process that determines the development of the CCP is contained within the CAAs. These are triggered when previously specified MDS 3.0 codes are selected for a resident. The interdisciplinary team members must then document underlying causes, contributing factors, and risk factors related to each CAA triggered, and explain why the resident issue was care planned or not. Documentation of this process is required; the format of this documentation is left to the SNF to determine [64].

Much of what is considered the standard of practice for CCP development is reminiscent of the early nursing care plans used as didactic tools [8]. The typical CCP is lengthy and contains standardized interventions found in textbooks. It is commonly physically located away from where direct care is actually provided [45]. Surveyors have come to expect that every medication, treatment, intervention ordered, or revision must be contained within the CCP to meet regulatory requirements. This results in duplication of records because such documentation is routinely included in the clinical, medication, and treatment records. This practice likely contributes to staff perceptions of the lack of utility and value of CCPs.

**Table 8:** Documents and staff responsibilities for the RAI.

Assessment	Decision-Making	Comprehensive Care Plan Development	Care Plan Implementation	Evaluation
Minimum Data Set/Other	Care Area; Assessment (not required for OBRA comprehensive assessments; required for Medicare PPS and OBRA comprehensive assessment)	CCP	Qualified staff identified on CCP or other qualified staff	Documentation by qualified staff identified on CCP or other qualified staff
Coordinated by RN with participation of clinical staff members; OR Conducted by assigned clinical staff members	RN coordinator certifies completion of CAA	Possible members: RN coordinator, other RN, licensed vocational nurse, nursing assistant, restorative nursing assistant, occupational, physical, speech therapists, dietician, resident, family member/resident representative, physician, medical director (for collaboration on current evidence-based standards of practice)	Qualified staff identified on CCP	Qualified staff identified on CCP

Note: Under 42 CFR 483.30 (Nursing Servicees), a SNF may be granted a waiver by the State to employ a RN who signs MDS 3.0 to certify its completion.

SNFs that are participating in culture change activities are more likely to include direct care workers, such as nursing assistants, in care plan meetings [62]. The assumption is that they are contributing to CCP development, but description of their training as interdisciplinary team members has not been explained. Some of these SNFs are creating “I-care plans” in which the CCP is developed using the language and perspective of the resident to document objectives, interventions, and timelines.

The state of current practice cannot be described accurately without discussing the role of the RN MDS Coordinator. Federal regulation 483.20 (h) stipulates that “a RN must conduct or coordinate each assessment with the appropriate participation of health professionals.” Table 8 displays other staff who may participate in the process. Although the RAI framework is complex, no specialized training is required for the RN MDS Coordinator and the health professionals providing services [7]. The only federal requirement for RN participation is that the RN designated as the MDS coordinator coordinates the completion of the MDS by other SNF staff completion. When she functions solely as the coordinator, she is not responsible for the accuracy of assessment data entered. As noted in Table 8, a State may issue a waiver to a SNF, enabling it to hire an RN specifically for signing and certifying completion of MDS 3.0. Estimates of the time required to complete the RAI processes vary widely, largely based on whether a research nurse or a working RN MDS coordinator is being timed [48]. This is an area of concern for RNs, given the limited presence of RNs working in SNFs. Researchers have reported that the majority of their time is spend in documenting care and other indirect care activities [65].

## 5. Recommendations for Practice and Research

### 5.1. Practice Recommendations

We used Prasad and Ioannidis’ (2014) conceptual framework for de-implementation of a standard of practice to guide us in developing micro and macro level practice recommendations [66]. They suggest that ubiquitous practices, supported by limited evidence, and that place a burden on the healthcare system, are priorities for de-implementation and establishment of an alternative evidence-based standard of practice. We believe that the extand standard of practice for CCP development as it relates to the CCP’s purpose and content would benefit from being revised based on evidence summarized in the scoping literature review.

#### 5.1.1. Micro-Level Recommendations for CCP Development

The CCP serves multiple purposes. It is used as a measure of regulatory compliance, a method of interdisciplinary communication and care coordination, and a repository of resident-specific care preferences, needs, and strengths. We recognize that there is a legitimate need for some type of CCP document. However, it is not practical or feasible to require the CCP to serve multiple purposes and necessarily reflect all interventions to be provided to a resident. For example, in an emergency, the competence of the SNF staff member, supported by existing policies and procedures, guides clinical behaviors. The clinical record is the document properly used to reflect the dynamic nature of services needed and used by the resident. Based on the findings of the scoping literature review, we recommend that the CCP’s purpose is realistically assessed, with an emphasis on operational implementation. As a standard of practice, the content of the CCP needs to be changed.

The sole purpose of the CCP ought to be that it is the centralized document used to coordinate interdisciplinary care by focusing specifically on those resident preferences, needs, and strengths that need to be known by every team member. This information is to be used in tailoring services provided to the resident in a manner that compliments his unique needs, strengths, and preferences. . Therefore, there is little benefit in documenting approaches and treatments that are found in other components of the clinical record. Similarly, descriptions of standardized approaches to interventions are not useful. Such information is best communicated in the form of a policy and procedure. Excluding this content will help to decrease the length and complexity of the CCP and increase its utility and value. This practice has the potential to support a more efficient and effective use of RN and interdisciplinary team member time.

The resident will benefit from a more tailored approach to CCP development. For example, he will have less need to repeat his preferences and particular needs to each staff person over time. Some resident specific information found in MDS 3.0 Manual Section F (Preferences for Customary Routine), could be included in the CCP, assuming that the preferences are actionable [1]. The direct care nurses could be taught how to share their observations of resident preferences, needs, and strengths with the interdisciplinary staff. Even if the practice of consistent assignments is used, there is benefit in documenting tailored information about the resident in one central document for all to access [67]. However, a resident and staff member may interact in ways that are unique to their relationship. These approaches do not need to be known by the entire team because they are unique to this dyad.

As important as CCP development may be, the CCP that is produced needs to be viewed as one of the many ways in which information about residents is exchanged and care is coordinated. Examples of other informal and formal ways of exchanging information include morning team meetings, handovers (e.g., change of shift report), huddles, and naturally occurring information exchanges among SNF staff that may or may not warrant inclusion in the CCP for all to know [60]. Development and testing of effective methods of communicating shift-specific information to individual staff members, such as that traditionally contained in a Kardex are needed. The carded or another method of sharing shift-specific information needs to be developed by SNF staff. They are in the best position to know the information to which they need easy access for each shift to perform their work effectively [50,67].

If the CCP is developed using these recommendations regarding its purpose and content, it still will not add value to the resident’s experience if it is not integrated into routine practices. Innovative, resident-specific, and useful CCPs are more likely to be implemented in SNFs having organizational cultures in which communication is open, staff are respected and valued, teamwork skills are taught and supported, and punitive managerial practices are absent [60,68]. Creating such a practice environment takes much effort and is challenging to sustain. As others have noted, the likelihood of achieving this will be influenced by SNF reimbursement levels, corporate human resource practices, and an investment in improving the leadership skills of SNF administrators and directors of nursing [69].

Implementing the recommended revisions in the purpose and content of the CCP will require courage. This undertaking is likely to engender fear because of the SNF’s potential risk of being cited for non-compliance with the extant standard of care. Innovation is difficult to achieve in a highly regulated practice environment. One approach that could be used to minimize fears is to define efforts in CCP development and implementation that are not the broadly accepted standard of practice would be to frame efforts as a quality improvement project. This could easily be supported within the mandated Quality Assurance Performance Improvement (QAPI) requirement [70]. Such an approach is would be evidence based, include participation of staff, and provide a practical mechanism for using knowledge to action framework in working towards making substantive revisions in extant standard of practice.

#### 5.1.2. Macro-Level Recommendations

Macro-level recommendations address how the CCP development requirement is measured; how staff is educated; and how RN staffing influences CCP development, implementation, and achievement of desired outcomes. Less emphasis needs to be placed on the CCP as a measure of regulatory compliance. Instead, the CAA documentation could be used as a measure of compliance. The CAA should reflect the quality of the decision-making processes used by the interdisciplinary team to develop a CCP. It is already a component of the RAI framework [64]. The requirement for the CAA process could be extended to include quarterly MDS 3.0 assessments.

We recommend training of state surveyors in evaluating compliance with care plan development and the care plan document from an evidence-based perspective. The intent is to reduce the focus on inclusion of standardized interventions that are documented elsewhere in the clinical record. Instead the focus needs to shift away from CCP content to evaluation of the staff’s implementation of care as defined by the resident’s documented preferences, needs, and strengths. We recognize that this would be more challenging to do compared with the current approach to evaluating compliance with CCP development. The CCP is important because it is a means to an end rather than an end in itself.

As previously noted, there is no requirement for RN MDS coordinator training or certification, such as that provided by the American Association of Nurse Assessment Coordination (AANAC) [71]. This is also the case for other members of the interdisciplinary team [72]. AANAC provider certification for RN MDS Coordinators, some of which would benefit other team members. Further, if interdisciplinary teamwork is a primary mechanism through which effective and efficient care is believed to be provided, it is reasonable to require a basic level of competence in this skill [58]. Nazir’s systematic review of randomized controlled trials of interdisciplinary interventions did find that interventions that focus on CCP team meetings, team communication, and coordination were consistent features of successful interventions [57].

The mandatory level of RN staffing levels needs to be addressed given that the RN is the only healthcare professional working in a SNF that has been specifically educated and licensed to coordinate care using critical thinking and problem solving skills. A higher level of RN staffing and a higher ratio of RNs to other members of the nursing skill mix have been associated with achieving higher levels of specific quality indicators [73].

CMS’ most recent proposal for federal NH regulatory changes does not include staffing or training requirements, including those of RNs and RN MDS nurses, except for nursing assistants and volunteers. Given this, we recommend that advocates continue their efforts to promote these changes by educating the public and SNF providers about the complex factors that influence the quality of SNF care, including CCP development, to facilitate adoption of our recommended changes.

#### 5.1.3. Research Recommendations

Further research is recommended to demonstrate the value-added by implementing revisions in the current standard of practice for CCP development, measurement of regulatory compliance, and surveyor and interdisciplinary team education. Little is known about how best to implement the RAI framework into the clinical routines of SNF staff. Further research on care plan development and operational implementation of the RAI process is needed to advance our understanding of how these practices add value to the resident’s and staff’s experiences. Studies that explore the relationship between SNF staff training in CCP development that is customized to the resident’s preferences, strengths, and needs, and the resident’s experience are needed.

A qualitative and mixed methods approach to studying CCP development is recommended as they have been effectively used to examine the complex process of CCP development and implementation within the RAI framework. In particular, complexity science [59,61], an adaptive leadership framework that complements principles of culture change [74], and information exchange system models [67] are well suited to use as conceptual frameworks in future studies of CCP. Theories of social networking will likely be beneficial to use in studying CCP development and implementation because of their emphasis on the influence of social relationships play in the workplace [59]. If we do not increase the evidence base describing best practices in these core components of SNF care delivery, we are less likely to be successful in having related, evidence-based CCP regulations implemented. Towards that end, there is a need for a rigorous systematic review of international practices related to CCP development within the RAI framework.

## 6. Conclusions

Multiple factors have affected the level of compliance with the regulatory requirement for CCP development within SNFs. The RAI framework was originally presented as a means of improving CCP development and implementation. Arguably, its primary purpose appears to be a standardized approach to data collection used for reimbursement, quality measurements, public reporting, and research. There is a need to refocus attention on the RAI framework’s use in CCP development and implementation. Empirical evidence provides support for revision of CCP development standards related to its purpose and content in an effort to add greater value to the resident’s experience because of development and implementation of the CCP. Timely consideration of these recommended changes in the existing standard of practice are needed because of the changed role of the SNF in the post-acute care continuum, the increased complexity of residents, and lack of improvement in federal standards for RN staffing. No federal requirements for RAI education among interdisciplinary team members and RN MDS coordinators have been made. In spite of these constraints, it is possible to redefine the standard of CCP development to produce a plan of care that is more likely to add value to the resident’s experience and quality of relationships with SNF staff.
